# Supplementary figures and images for: Particulate matter resuspension from simulated urban green floors using a wind tunnel-mounted closed chamber
Source: PeerJ. 2023 Feb 8;11:e14674. doi: 10.7717/peerj.14674 (PMC9921991; doi:10.7717/peerj.14674)

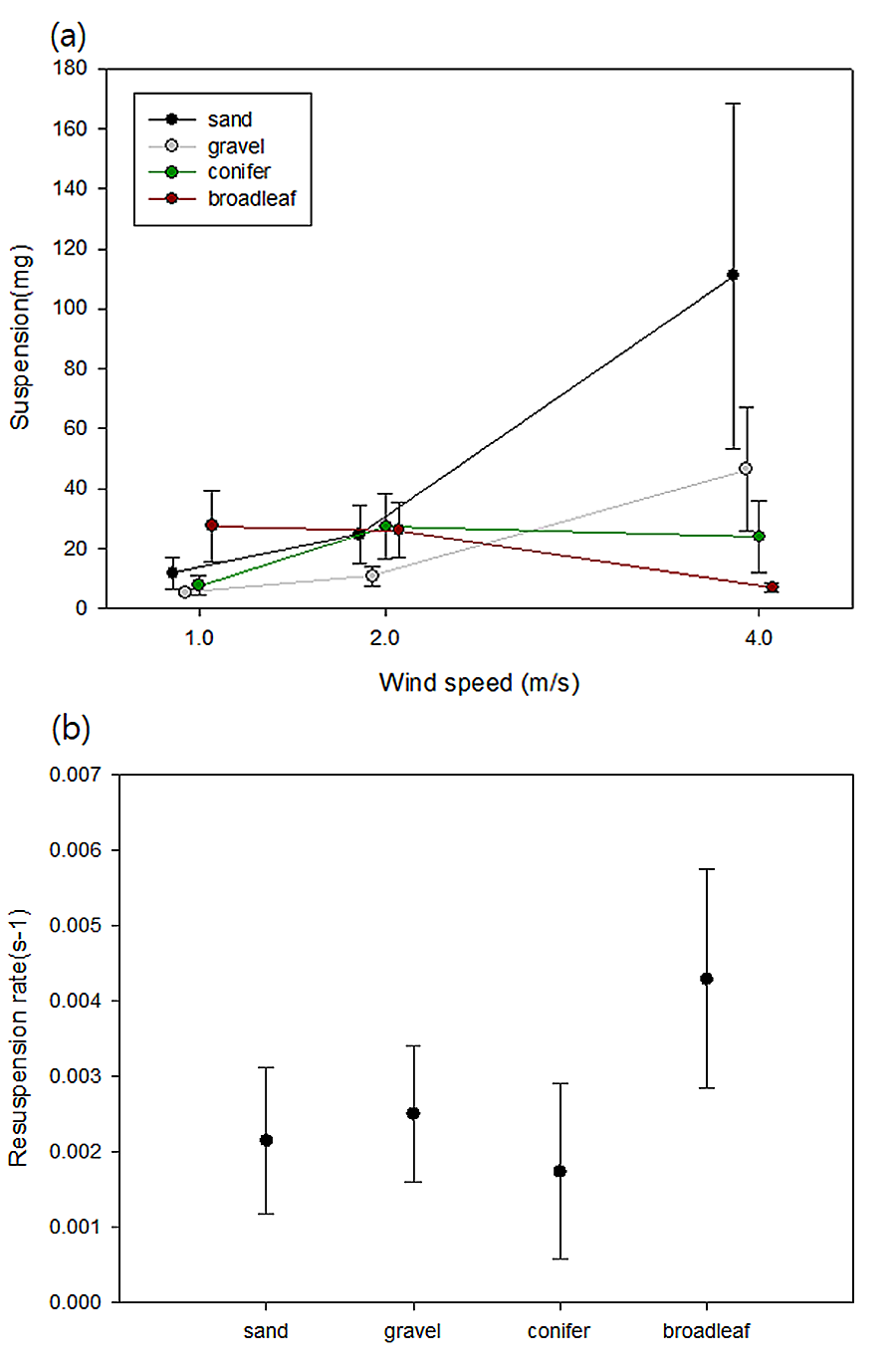

Supplement: Supplemental Information 3 — The wind speed conditions varied by 1, 2, 4 m s-1. (A) The amount of background suspension, (B) resuspension rates at 4 m s-1 wind speed. The background suspension amount and resuspension rates were different only at 4 m s-1. [file peerj-11-14674-s003.png]
